# Preparation of core-shell structure KClO_4_@Al/CuO Nanoenergetic material and enhancement of thermal behavior

**DOI:** 10.1038/s41598-017-03683-z

**Published:** 2017-06-16

**Authors:** Fan Yang, Xiaoli Kang, Jiangshan Luo, Zao Yi, Yongjian Tang

**Affiliations:** 1 Institute of Modern Physics, Fudan University, Shanghai, 200082 China; 2 Research Center of Laser Fusion, China Academy of Engineering Physics, Mianyang, 621900 China; 3 Joint Laboratory for Extreme Conditions Matter Properties, Southwest University of Science and Technology, Mianyang, 621900 China

## Abstract

In this paper, a solvent/non-solvent synthetic approach has been utilized in preparing a new nanoenergetic material KClO_4_@Al/CuO by coating Al/CuO nanocomposites particles with a layer of nanoscale oxidizer KClO_4_. The coating process and mechanism are discussed. The composites of Al/CuO are uniformly mixed by mechanical ball milling process and CuO acts as a catalytic metallic oxide. The ternary mixtures KClO_4_@Al/CuO were characterized by X-ray diffraction (XRD) and the results reveal that after ball-milling and chemical synthesis process, the phase compositions haven’t changed. Scan electron microscopy (SEM) images show that these energetic nanocomposites consist of small clusters of Al/CuO that are in intimate contact with a continuous and clear-cut KClO_4_ layer (100–400 nm). In a Scanning transmission electron microscopy (STEM) elemental map, high K/Cl intensity on the perimeter of the nanoparticles and high Cu/Al content in the interior powerfully demonstrated the KClO_4_@Al/CuO core-shell nanostructure. Electrical ignition experiments and pressure cell test prove that these nanoenergetic composites are more sensitive to ignition with much higher burning rate than traditional formulations (conventional counterparts). To quantify the enhancement of thermal behavior, Thermogravimetry (TG) and Differential scanning calorimetry (DSC) were performed and the results show that the burning rate of these energetic nanocomposites nearly tripled.

## Introduction

Energetic materials are a class of compounds or mixtures that contain an explosive group or contain an oxidizer and a fuel with high mount of stored chemical energy that could be released. Typical classes of energetic materials are e.g. propellants, explosives, and pyrotechnics. Basically, there are two kinds of traditional EMs: (1) monomolecular EMs (contain the oxidizer and fuel components in one molecule); (2) composite EMs (mixtures which are produced by physical mixing the oxidizer powders and fuel powders)^1^. These composite EMs exhibit high energy density and mixing the fuel and oxidizer powders in stoichiometric ratio are able to obtain maximum energy density. Compared to monomolecular EMs, the energy release rates of traditional composite EMs are slower because the mass transport rate is limited by the large grain size of the reactants and inhomogeneous distribution of components. To achieve a chemical-kinetically controlled ignition, a fascinating class of high-energy release rate materials called metastable intermolecular composite (MIC) have attracted extensive interest and attention over the past decade^2–4^. The MIC also called inorganic energetic composite combining metal oxidizer and metal fuel undergo a rapid solid-state redox reaction that is very exothermic (almost two times that of the best monomolecular EM). Here we define MIC as any composite energetic material utilizing nano-scale energetic constituents. The use of nanoscale material and the intimate mixing of constituents on nano-scale reduce the mass transport limitations between the fuel and oxidizer making these nanoscale EMs (nEMs) attractive alternatives to monomolecular structures. The most widely used materials of the possible MIC formulations are nano-Aluminum (Al) combine with nano-size metal oxidizers. Among a large number of possible oxidizers^5^, only a few have been investigated: Fe_2_O_3_, MoO_3_, KMnO_4_, CuO, NiO, MnO_2_, WO_3_, SnO_2_, and SiO_2_. It has been demonstrated by the researchers of LANL that nano-clusters of MoO_3_ */*Al MIC (with particle diameters of 20–50 nm) burn 1000 times faster than macroscale thermite material^6^. Numerous nanoporous oxidizers for nanoenergetic materials have been synthesized by using sol-gel chemistry^3, 7^, and intermixing the fuel and oxidizer required a secondary step of ultrasonic mixing which relies on a random mixing process. Zachariah realized the directed assembly between fuel and oxidizer by electrostatically enhanced assembly method^8^. The reactivity of energetic nanocomposites has been enhanced by enhancing the interaction of fuel and oxidizer and minimizing fuel-fuel and oxy-oxy interactions.

In addition, because of its mild phase transition, moderate sensibility and fast ignitability, potassium perchlorate (KClO_4_) is widely used for applications as a oxidizer involving propellants and pyrotechnics for a long period^9–11^. Compared with three most widely used oxidizers MoO_3_, CuO, and Fe_2_O_3_, it has the highest reaction enthalpy with fuel Al (which are calculated by HSC chemistry 5.1). Thermodynamic characteristics of Al combustion with the above four oxidizers are listed in Table 1. The reaction between KClO_4_ (Kp) and Al could liberate a large amount of energy. Moreover, it was able to emit strong light contain specific lines in the visible-near infrared spectrum which exist various potential and promising applications in pyrotechnics and optical field^12–15^. Compared with other oxidizers, Kp is difficult to nanocrystallization by conventional sol-gel chemistry. Recently Pourmortazavi has synthesized Kp nanoparticles via spraying in non-solvent process^16^. Besides many papers have investigated the catalytic effects of metal oxides on the decomposition of Kp, among them tenorite (CuO) containing transition metal cations with partially filled *d*-orbitals has the highest activities. The Cu^2+^ in metal oxides tend to attract the unshared electron pairs of oxygen atoms in ClO_4_^−^. This interaction can weaken the oxygen-chlorine bond in ClO_4_^−^ and thus can facilitate the reaction between Al and Kp. Cu^2+^ (*d* ^9^) with partially filled *d*-orbitals are relatively small in size since there is less electrical shielding. Therefore, they are more inclined to attract the extra unshared electron pairs to form ligands with the oxygen atom in ClO_4_^−^^17–23^.

**Table 1:** Thermodynamics of Al combustion in different oxidizers.

Reaction	adiabatic flame temp. (K)	ΔH(kJ/mol of Al)
2Al + Fe_2_O_3_	3198	−425
2Al + 3CuO	3794	−604
2Al + MoO_3_	3812	−465
3Al + KClO_4_	3330	−840

**Figure 1:**
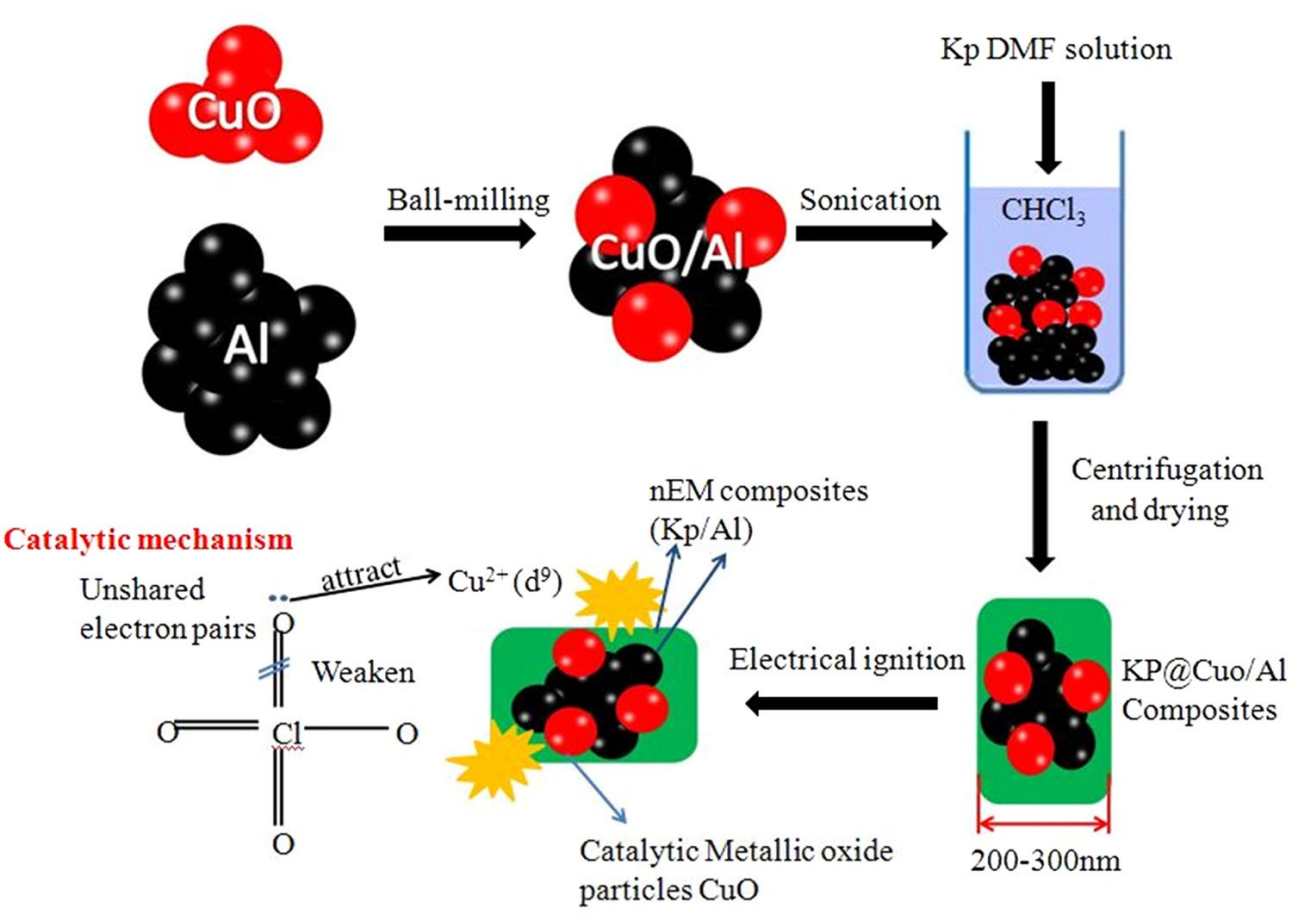
Schematic of the fabrication and electrical ignition of Kp@/Al/CuO nanocomposites.

The main aim of this work was preparation of a new MIC formulation of nano-Al combines with Kp nanoparticles using CuO as catalyst via ball-milling and solvent/non-solvent synthetic approach. The ternary mixtures are composed of Al nanoparticles as the fuel, Kp nanoparticles as the oxidizer, and CuO as the catalytic metallic oxide. CuO was chosen as a accelerator to generate a high amount of heat which could be released between CuO and Al, leading to a more convenient reaction between Al and Kp in the process. The reactivity of the MIC is about 3 times higher than traditional formulations (their conventional counterparts). Kp nanoparticles continuously distributed over the surface of Al/CuO particles to form core-shell structure. The Core-shell structure nanocomposites which rely on the directed assembly between fuel and oxidizer react vigorously with much higher burning speeds and much faster energy release velocity than those produced by mechanical ball milling or ultrasonic mixing process because of the decrease of mass transfer distance and the increase of effect contact surface area of reactants. Besides, after the coating layer was fabricated, it could prevent the further oxidation of Al during storage. A schematic of the fabrication and electrical ignition of Kp@Al/CuO nanocomposite is presented in Fig. 1.

In this paper, firstly, the fuel and catalyst were uniformly mixed by ball-milling experiments. Then a series of batch experiments were conducted to study the effects of the concentration of oxidizer solution, solvent identification, and temperature difference between solvent and non-solvent on the scale and morphology of coating particles. The optimal conditions and coating mechanism to prepare the nanocomposite particles were discussed. Finally, the core-shell structure of the nanoenergetic materials were reported and an example of the reactive characteristics were shown.

## Results and Discussions

**Figure 2:**
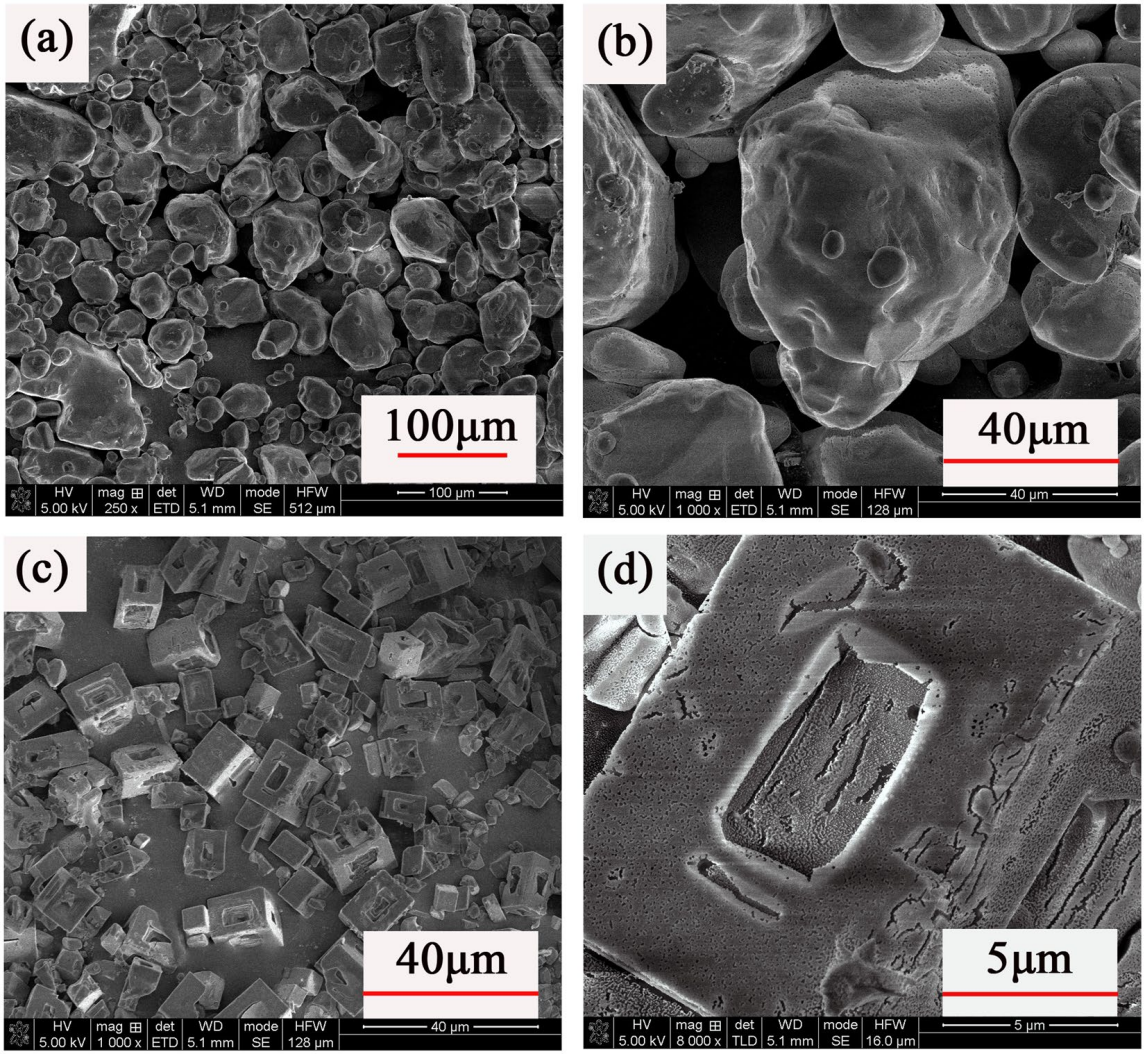
(**a**), (**b**) SEM images of raw Kp particles at 250x, 1000x (**c**), (**d**) SEM images of Kp particles synthesized by the condition of Kp water solution(1% w/v) added to 200-proof ethanol at 1000x, 8000x.

In the experiment, we explored the optimal conditions to synthesize nano-scale Kp particles without adding the nucleation core Al/CuO composites (homogeneous nucleation process). 6 ml of Kp water solution were simultaneously added to 36 ml 200-proof ethanol at 60 °C and ultrasonicated. The temperature difference between solvent and non-solvent was 40 °C. The SEM images of raw Kp particles and the synthesized Kp particles are shown in Fig. 2. The raw Kp particles have an irregular shape with wide particle size distribution. After no-solvent process, the scale of Kp particles has decreased and the size distribution become uniformly, but the scale of Kp particles remain micron size. It could be seen that the crystals form a porous network of particles by magnifying the SEM images of the synthesized Kp particles. Obviously, the porous structure of high surface area would lead to the enhancement of thermal behavior.

**Figure 3:**
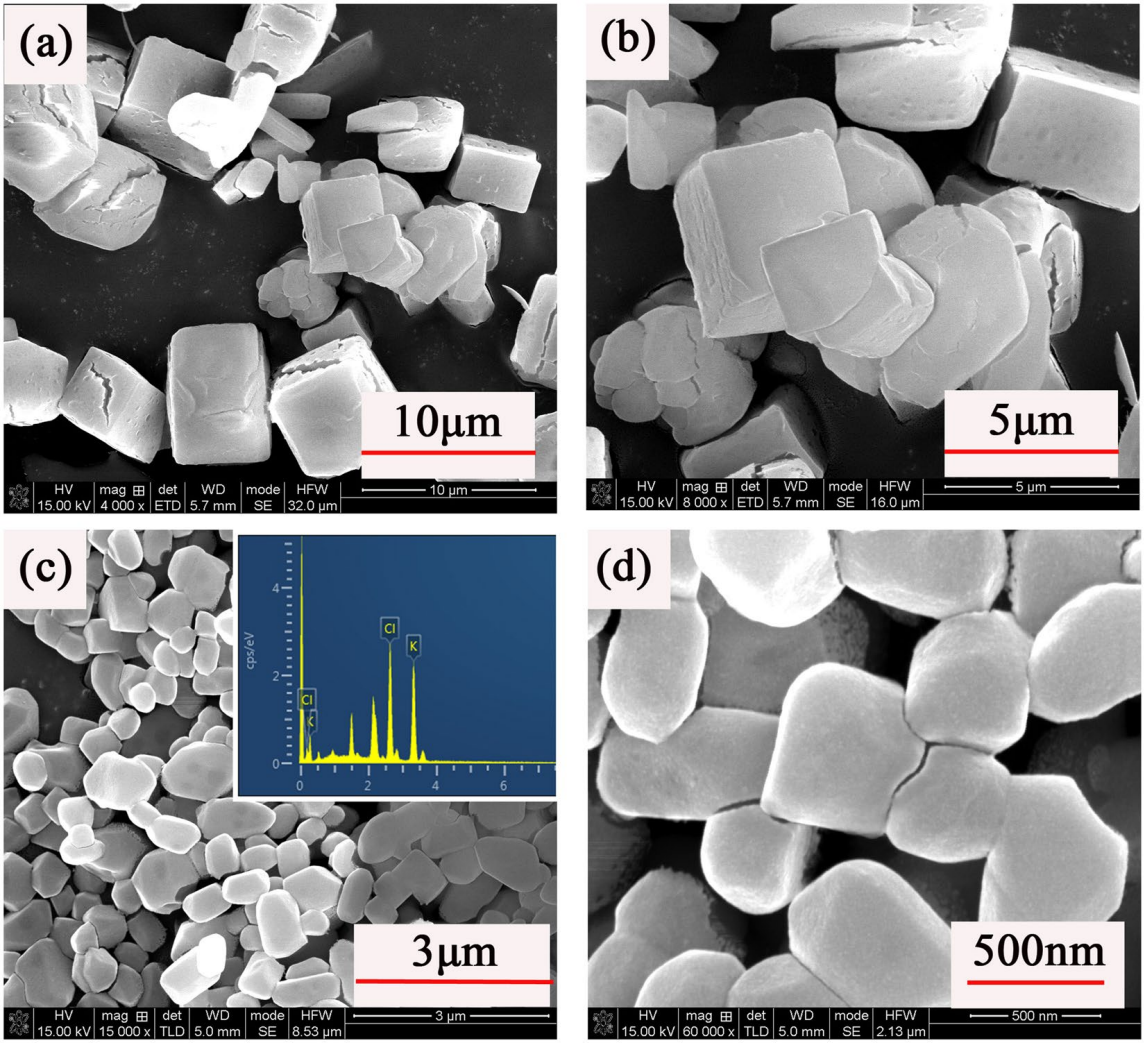
(**a**), (**b**) SEM images of Kp particles synthesized by the condition of Kp DMF solution (1% w/v) added to CHCl_3_ at 4000x, 8000x (**c**), (**d**) SEM images of Kp particles synthesized by the condition of Kp DMF solution (3% w/v) added to CHCl_3_ at 15000x, 60000x.

Then we keep the experimental condition except changing the solvent/non-solvent identification to Dimethyl formamide/Chloroform (DMF/CHCl_3_), the scale of Kp particles hasn’t decreased and the porous structure disappear. The solubility of Kp in DMF is much higher than that in water and it increases with temperature in a manner of power law. By increasing the concentration of Kp DMF solution from 1% w/v to 3% w/v at 80 °C, the particle size has effectively decreased from micron size to nanoscale. The SEM and EDX images are shown in Fig. 3. The size of Kp particles synthesized under the condition decreased below 500 nm.

**Figure 4:**
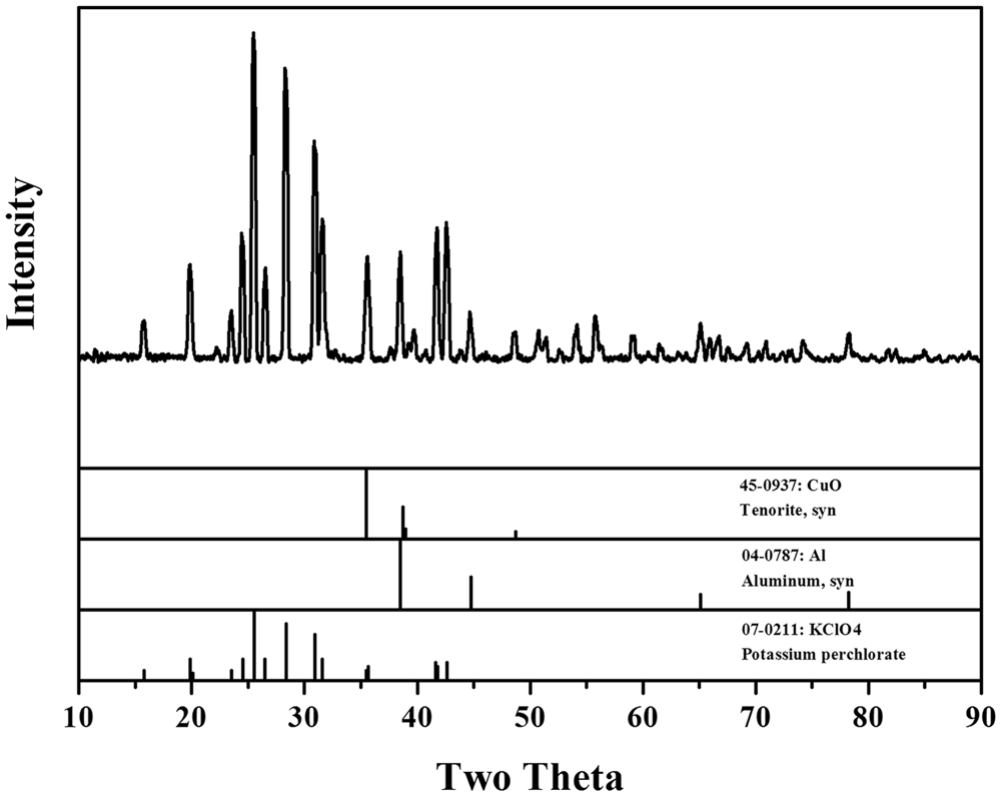
X-ray diffraction patterns of ball milling Al/CuO composites coated with Kp.

Finally, we utilized the optimal conditions to synthesize the ternary nanocomposite particles with adding the nucleation core. The coating process and nucleation mechanism are discussed. The coating process is actually heterogeneous nucleation process. The Kp molecules are uniformly dispersed in the solvent. These molecules collide with each other during high speed stirring. After adding high temperature Kp solution to nonsolvent, Kp molecules precipitate on the surface of Al/CuO to form nucleation. Because of the temperature difference and competition relationships between solvent and nonsolvent, the Kp molecules in liquid phase continuously diffuse to the surface of Al/CuO to make the nucleation grow up to form heterogeneous coating. Based on Lamer’s nucleation theory^24^, the increase of super-saturation degree could benefit the increase of crystallizer nucleation velocity and the decrease of crystallite dimension. It is confirmed by our experiment, with the increase of super-saturation degree by increasing the concentration of Kp solution, temperature difference between solvent and non-solvent, the medium particle size of Kp has effectively decreased. Clearly, the crystallite dimension is smaller in heterogeneous nucleation process than that in homogeneous nucleation process. The optimal conditions are described in experimental section: 6 ml of Kp DMF solution (3% w/v) were simultaneously added to 36 ml Al/CuO CHCl_3_ emulsion and ultrasonicated, After the fast evaporation of the solvent, the coated samples are fabricated. X-ray diffraction patterns of the coated samples (ball milling Al/CuO composites coated with Kp) are showed in Fig. 4. The main components identified were metallic Al, CuO and oxidizer Kp without any new constituent appearing. They are the expected products if the thermite reaction hadn’t occurred during the process of ball milling and chemical synthesis.

**Figure 5:**
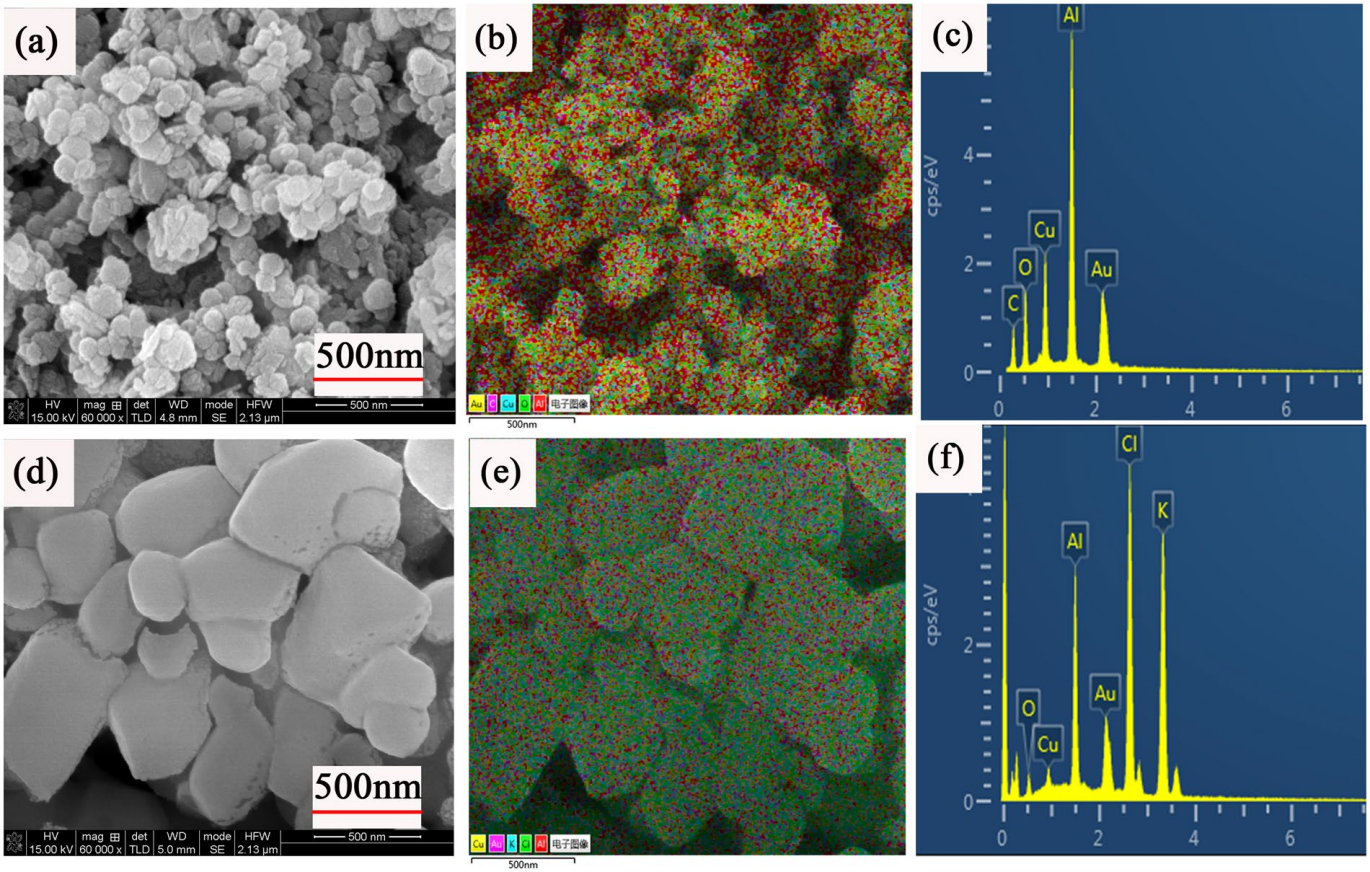
(**a**) SEM (**b**) Surface elements distribution (**c**) EDX images of ball-milling Al/CuO particles (**d**) SEM (**e**) Surface elements distribution (**f**) EDX images of Kp@Al/CuO nanocomposite particles.

Figure 5(a) and (d) present scan electron microscopy (SEM) images of the particles of unprocessed and coated ball-milling Al/CuO by solvent/non-solvent approach. The ball-milling Al/CuO particles are spherical and disperse evenly. An obvious clear-cut particle layer is continuously distributed over the surface of Al/CuO particles, the coated particles are quadrilateral with a size distribution range from 100–400 nm. Figure 5(b) and (e) show distribution of the elements on the surface of nanocomposite particles. It could be seen that the Cu and Al elements are uniformly distributed across the scan area after ball-milling, a continuous coating layer containing K and Cl elements which proved to be the oxidizer Kp particles are distributed all over the surface of ball-milling Al/CuO. Besides, by the approximate calculation of surface elements content which are shown in Fig. 5(c) and (f), the content of CuO is little and the weight ratio of Al/Kp components is nearly 30/70 which is near the stoichiometric weight ratio equal to 34.2/65.8.

**Figure 6:**
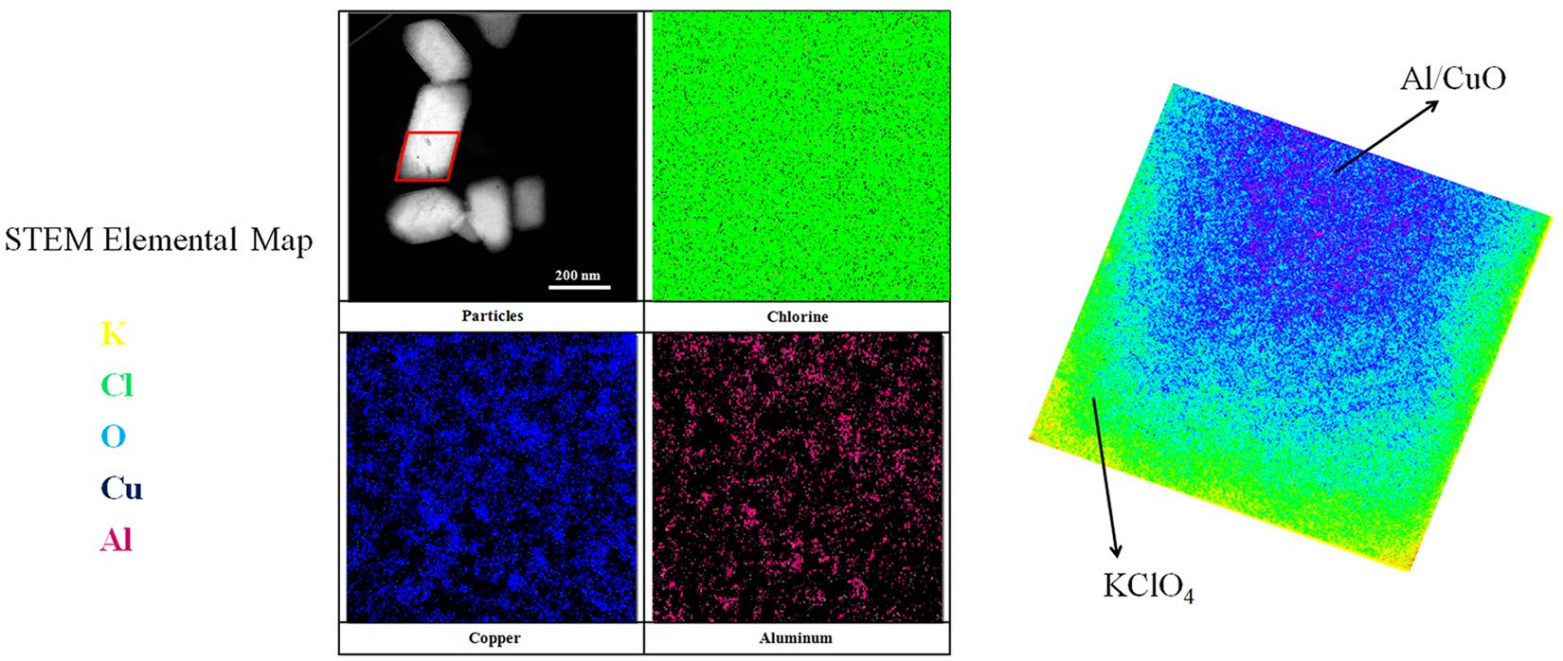
STEM elemental map showing the distribution of Cl, Cu, and Al in the particles.

Scanning transmission electron microscopy (STEM) elemental mapping on the nanocomposite particles have been obtained to examine the core-shell structure and are shown in Fig. 6. In a STEM map, the electrons scan a square area of a particle to identify the different elements present in the view. The density of the points represents the content of different elements. It could be seen clearly that the surface of the nanocomposites are clear-cut and the shape of it is consistent with Kp particle (quadrilateral). A high intensity on the Chlorine map colored by green all over the particles suggests that the surface component of shell is Kp. The copper colored by blue and aluminum colored by pink are uniformly distributed throughout the particle which suggest the presence of Al/CuO as a core in the Kp particle shell.

**Figure 7:**
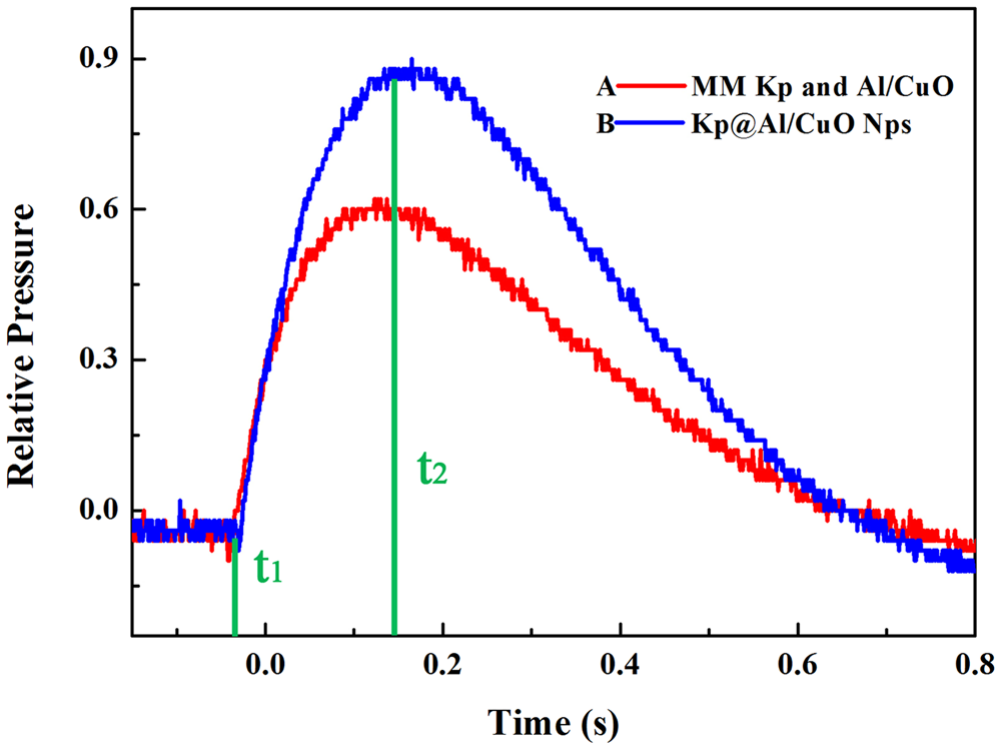
Pressure cell test on A: MM Kp and Al/CuO B: Kp@Al/CuO Nps.

**Figure 8:**
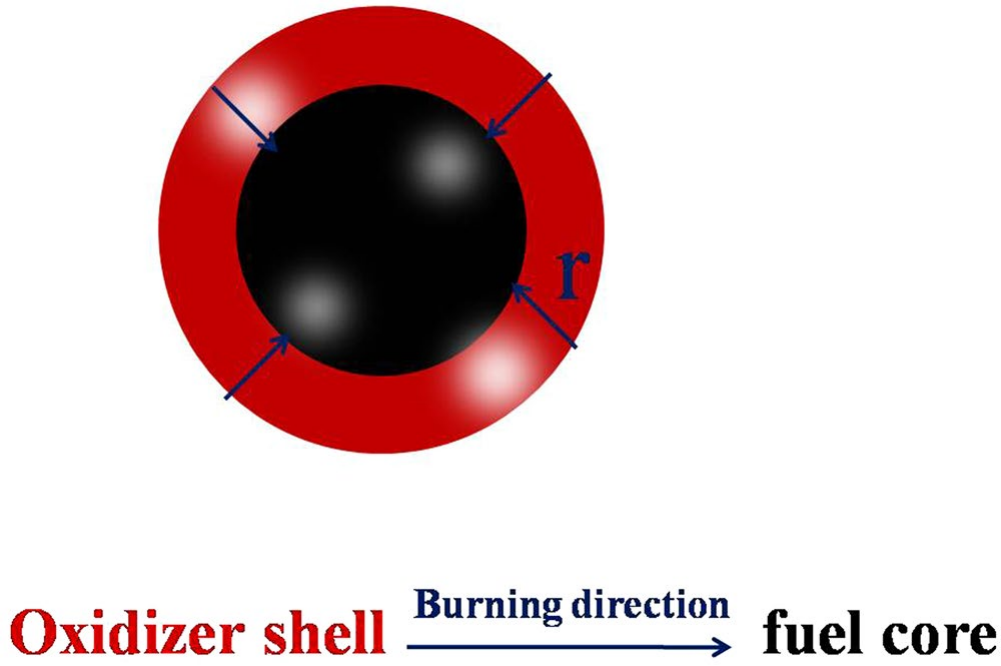
Schematic of the combustion of a nanoparticle coated with a thin oxidizer.

Finally, to assess the suitability of Kp@Al/CuO nanocomposite particles as propellants or explosives, pressure cell tests were performed on the mechanical mixing (MM) Kp and Al/CuO as reference sample (the used raw Kp particles were shown in Fig. 3), and Kp@Al/CuO nanoparticles (Nps). About 50 mg of the mixture was pressed into pellets at 1.5 Mpa pressurization and ignited in a sealed chamber of constant volume with a Ni-Cr wire, and the chamber pressure was measured as a function of time. The observed pressurization rate of Kp@Al/CuO Nps presented in Fig. 7 is more dynamic than the traditional MM Kp and Al/CuO mixtures, which is 1.6 times higher. The proportion of oxidizer and fuel are almost the same except the mixing process and the scale of oxidizer. The intimate mixing of components of core-shell structure KClO_4_@Al/CuO Nps on nano-scale reduce the mass transport limitations between the fuel and oxidizer thus the burning rate is much higher than the reference sample. To explain that, we consider a thin oxidizer layer coated on a spherical fuel particle (like Al) which burns from the perimeter to the interior, as shown in Fig. 8 ^1^. The diameter of the particle *d* decreases as the time *t* by the equation: *d* ^*2*^ = *r* × *t/*π with *r* as the burn rate (mm^2^ */*s). Therefore, with the low limit *r* = 1.5 mm^2^ */*s, particles of 200 nm in diameter would be consumed in 0.04 *μ*s. Compared to mass diffusion time of a Al particle with 100 nm in diameter (hundreds of Microseconds), This time is adequately short that the mass diffusion process is negligible in this situation.

**Figure 9:**
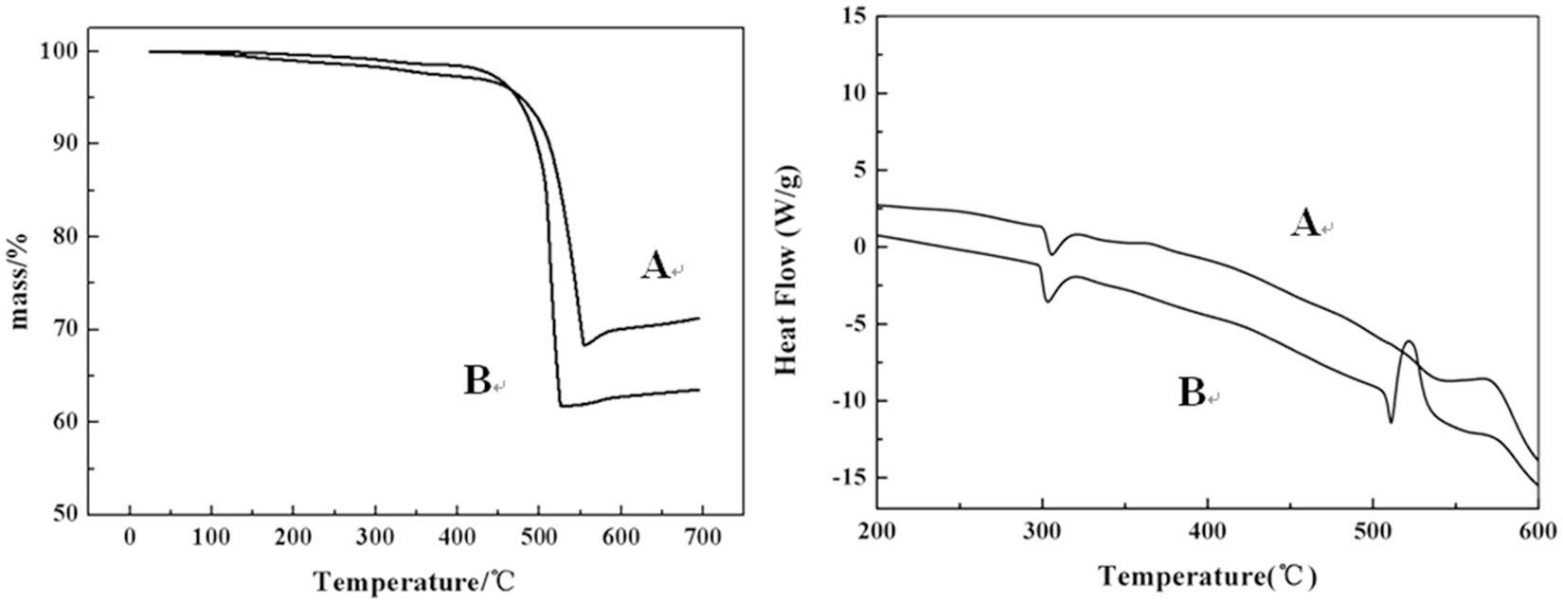
TG-DSC curve of the samples at heating rate of 10 °C. min^−1^ in air atmosphere (**A**) MM Kp and Al/CuO (**B**) Kp@Al/CuO Nps.

To further quantify the improvement of thermal behavior, thermal analysis using TG and DSC was performed on both A. MM Kp and Al/CuO and B. Kp@Al/CuO Nps which are shown in Fig. 9. The phase change of Kp from rhombic to cubic structure occurs at 301 °C with an endothermic peak. The endothermic peak above 500 °C followed by a sharp exothermic peak corresponded to the melting of Kp and reaction between Al and Kp:

1 $$3{{\rm{KClO}}}_{4}+8{\rm{Al}}\to \,4{{\rm{Al}}}_{2}{{\rm{O}}}_{3}+3{\rm{KCl}}$$

The melting point of Kp is 575 °C. The reaction temperature of sample A is around the melting point 575 °C. The reaction temperature of sample B is much lower. To Produce solid-state pyrotechnic reaction, solid fuel and oxidizer atoms in direct contact should be enough and we need to provide the atoms with the required activation energy. For MM Kp and Al/CuO ternary mixtures, the number of Kp and Al atoms that are in direct contact with each other is normally quite small because of large grain size and the inhomogeneous distribution of components by mechanical mixing. However, if the oxidizer Kp melts to flow around the surface of the Al particles at the melting point, much more number of atoms will get in contact with each other. Therefore, with enough atoms of energies exceeding the activation energy barrier in contact with each other, the ignition could occur at the temperature of the melting point like sample A. This also means that the ignition can occur at a lower temperature below the melting point like sample B. Clearly the nanocomposite structure with intimate contact of components led to considerable effects of reduction in the reaction temperature in Al–Kp nanothermite system. Besides, Kp@Al/CuO nanocomposite particles synthesized via solvent/non-solvent approach had burning rates that were 3 times higher than those produced by conventional mechanical mixing of solid oxidizers and fuels with catalyst because of the decrease of mass transport distance^14^.

However, the complete reaction between Kp and Al would result in less than 5% mass losses by approximate calculation according to Eq. (1), whereas the observed loss was about 35–40%. A main reason is the oxidation of Al in air, therefore the decrease of effective Al content make the thermite reaction of Al with Kp incomplete. The Kp decompose according the following equation:

2 $${{\rm{KClO}}}_{4}\to {\rm{KCl}}+2{{\rm{O}}}_{2}$$

Although the thermite reactions between Al and Kp were incomplete because of the oxidation of Al in air, after the coating layer was fabricated, it could prevent the further oxidation of Al during storage. The shelf life could be significantly extended.

## Conclusions

This paper reports on our efforts to employ solvent/non-solvent chemistry to prepare a new MIC formulation (nano oxidizer particles composed of Kp coated on ball-milling Al/CuO) that may have application to nanoenergetic compositions. We have shown that with the increase of super-saturation degree by increasing the concentration of Kp solution, temperature difference between solvent and non-solvent, the particle size of nanocomposites particles have decreased from micron size to nanoscale. Characterization of these particles has shown that the nanocomposites particles within a size range of 100–400 nm formed a continuous and complete coating layer. Ignition and pressure cell tests shows that the nanocomposites react vigorously with much higher burning speeds and much faster energy release velocity because of the decrease of mass transfer distance and the increase of effect contact surface area of reactants. By employing solvent/non-solvent chemistry route, one may be able to rapidly and reproducibly obtain the core-shell nanocomposite energetic material with high purity and controlled fuel and oxidizer balances. The new MIC formulation is a potential and promising material for high-temperature engineering applications as propellants, explosives, and pyrotechnics.

## Experimental Details

The fuel nano-Al used for this work was commercially available from Aladdin industrial Corporation with average particle size ~50 nm. Kp (300 mesh) was purchased from kermel (Tianjing, China). Highly pure CuO powders were prepared by HWnano (Wuhan, China) technology with particle size 50–80 nm. DMF, CHCl3, and 200-proof ethanol were all analytical grade from the Guanghua technology (Guangdong, China) and used as received.

The Al/CuO composites were synthesized by ball milling experiments which carried out by a planetary ball milling apparatus. The nanocomposite powders were prepared under argon gas via batch-type ball-milling method (regularly stopped 1 h after 2 h of ball milling) with a rotational speed of 150 r/min. Based the work of zhang *et al*., CuO was mixed Kp in a molar ratio of 4%, corresponding to a ratio of one mole of metal cations to 24 mole of Kp^23^. CuO was mixed with Al in a molar ratio of 2% by approximate calculation. 500 mg Al/CuO mixtures were added into a 200 ml stainless steel container in a vacuum glove box.

The Al/CuO composites were coated with Kp by the method of integrating solvent/non-solvent process, A series of experiments have been done to obtain the optimal conditions under which nano-scale core-shell structure are fabricated and the fuel and oxidizer balances are strictly controlled. The optimal conditions of the experimental process were as follows: CHCl_3_ was used as the non-solvent. 10 mg ball milling Al/CuO nanocomposites were added to 36 ml CHCl_3_ and ultrasonicated for 15 min to obtain a black emulsion at 20 °C. 6 ml of Kp DMF solution(3% w/v) were heated to 80 °C and simultaneously added to the emulsion. The solution was stirred at the speed of 1000 r/min. Until the solvents are completely removed, the temperature drops to room temperature at a cool rate of 10 K/min. After centrifugation, filtration, and evaporation in vacuum at 70 °C for 4 hours, the Kp@Al/CuO nanocomposites are fabricated. The coating materials were completely dried and weigh 35 mg. The content of Kp is about 25 mg by calculation assuming no mass loss of Al/CuO nanocomposites during the whole experiments. The weight ratio of Al/Kp is approximate 30/70 near the stoichiometric weight ratio equal to 34.2/65.8.

To assess the scale of the composite particles, particle size distributions were measured by a laser particles analysis system. X-ray diffraction experiments were carried out on the powder samples to check the phase compositions utilizes CuK_α_ radiation. Scanning electron microscopy (SEM) and energy dispersive spectrometry (EDS) were performed on the nanocomposite particles to observe surface morphology and detect elements distribution. Scan transmission electron microscopy (STEM) elemental map was operated at 200 kv to demonstrate core-shell nanostructure. The thermal behavior was analyzed by TG and DSC (4 mg of powder samples were heated from 25 °C to 700 °C at 10 °C/min heating rate in air). The nanocomposite particles are pressed into pellets at 1.5 Mpa pressurization with a theoretical density which could extremely facilitate the burning behavior. Pressure cell tests were performed on the pellets to monitor the chamber pressure as a function of time.
